# Microfluidic Packaging Integration with Electronic-Photonic Biosensors Using 3D Printed Transfer Molding

**DOI:** 10.3390/bios10110177

**Published:** 2020-11-14

**Authors:** Christos Adamopoulos, Asmaysinh Gharia, Ali Niknejad, Vladimir Stojanović, Mekhail Anwar

**Affiliations:** 1 Department of Electrical Engineering and Computer Science, University of California Berkeley, Berkeley, CA 94720, USA; agharia@berkeley.edu (A.G.); niknejad@berkeley.edu (A.N.); vlada@berkeley.edu (V.S.); 2 Department of Radiation Oncology, University of California San Francisco, San Francisco, CA 94158, USA; Mekhail.Anwar@ucsf.edu

**Keywords:** integrated photonics, microfluidics, packaging, photonic biosensors, optical resonators, multiplexed sensing, 3D printing

## Abstract

Multiplexed sensing in integrated silicon electronic-photonic platforms requires microfluidics with both high density micro-scale channels and meso-scale features to accommodate for optical, electrical, and fluidic coupling in small, millimeter-scale areas. Three-dimensional (3D) printed transfer molding offers a facile and rapid method to create both micro and meso-scale features in complex multilayer microfluidics in order to integrate with monolithic electronic-photonic system-on-chips with multiplexed rows of 5 μm radius micro-ring resonators (MRRs), allowing for simultaneous optical, electrical, and microfluidic coupling on chip. Here, we demonstrate this microfluidic packaging strategy on an integrated silicon photonic biosensor, setting the basis for highly multiplexed molecular sensing on-chip.

## 1. Introduction

Label-free assays provide real time information for molecular analysis without requiring labeling of the target analytes that require customized molecular labels, multiple additional steps, and preclude monitoring real-time binding kinetics useful for characterizing molecular interactions. This makes them ideal for point-of-care assays. However, to date, label-free assays have been hindered by the need for complex, bulky optics precluding both mass production and miniaturization and remain confined to special laboratory settings [1,2,3]. In an effort to address these dual challenges, silicon photonic biosensors—fabricated using techniques based on integrated circuit manufacturing—have shown increasing promise in monitoring label-free molecular interactions through the evanescent field interaction of light, tightly confined to a waveguide, with the surrounding optical environment. In particular, one such structure where light of a specific wavelength is trapped in a ring (called a micro-ring resonator or micro-ring resonator (MRR)-based photonic devices), has been able to detect a wide range of molecular concentrations of different target analytes ranging from femtomolar to micromolar by leveraging high transducer sensitivity and small transducer area [4,5,6,7,8,9,10,11,12,13]. However, one of the key challenges towards efficient and diverse multiplexed sensing of multiple biomarkers is the fabrication of fluidic delivery devices to interface with the extraordinary density and degree of integration of arrays of micrometer-scale MRRs, while allowing for both optical and electrical coupling to the integrated circuit, which is often on a millimeter-square scale. Microfluidic interfaces with silicon photonic chips require (a) regular surfaces to form a seal and prevent leaking, (b) optical clarity for alignment, (c) vertical cylindrical interconnections with an aspect ratio of 5, which is defined as the ratio of their height to their width, to connect microfluidic layers with a high channel density (100 μm–200 μm channel width and edge to edge channel spacing), (d) access areas allowing fiber optics to couple light into the system, and (e) a mechanism to interface multiple microfluidic inputs to millimeter-scale chips with densely packed sensor arrays.

The current approaches to meet these constraints often require custom packaging techniques in order to accommodate centimeter scale microfluidics [14] or complex post processing following conventional multilayer soft lithography [15]. The bonding of microfluidics to chips typically requires a broad, flat surface in order to provide an adequate seal and structural support for the microfluidics. A key challenge arises with the small chip size (square millimeter-scale), constrained by the cost of manufacturing large (square centimeter-scale) chips. Large chip surfaces cannot be manufactured due to the high cost per unit area of manufacturing silicon-based devices. Thus, many small sensor elements are packed together in tightly spaced arrays. This makes it conventionally challenging to interface with standard microfluidics and inlet-outlet tubes, which are on the order of 5 mm diameter (needed to interface with standard pumps and sample sources), since they do not fit on the area of a traditional chip. While there are many techniques to create a flat surface for attaching microfluidic devices [14], we require a method to interface millimeter scale microfluidics with a small chip. We create a small interface microfluidic chip that securely bonds on the silicon photonic chip [15,16]. By doing this, we eliminate the need for larger chip areas that would significantly increase the cost. This bottom microfluidic layer then interfaces through vertical interconnections with another layer that expands microfluidic dimensions, so that they can interface with standard sized microfluidics and macro-scale tubing over a larger surface area.

This multilayer interface approach requires precisely aligned microfluidics at two different scales. Creating each layer via photolithography traditionally offers the greatest precision in channel dimensions and surface finishes, but it requires complex processing to create high-density vertical interconnections and align multiple layers [17]. A hybrid of soft lithography and laser micromachining can be used to address these challenges but requires expensive and specialized equipment [15]. Additive manufacturing can greatly reduce the complexity in fabricating such multilayer devices through 3D printed transfer molding wherein each layer is cast on a printed mold. Despite the promise of 3D printing, the fabrication of molds for microfluidics has been historically limited by the inability to produce the smooth surface finishes—necessary for both visualization through the microfluidic layers as well as proper sealing—and minimum feature sizes of photolithography, and has thus been limited to centimeter scale microfluidics [18].

Commercial printing technology has now crossed the resolution threshold in order to overcome surface finish, feature size, and aspect ratio constraints in designing millimeter scale microfluidics. Leveraging these advances, we demonstrate a simple, highly scalable, and versatile multilayer microfluidic device for CMOS integration created by 3D printed transfer molding and bonding. This packaging architecture can enable multiplexed analyte sensing without requiring large chip surfaces and complicated fabrication techniques, while it ensures a robust interface between the photonic sensors and the fluidic samples.

## 2. Materials and Methods

**Figure 1:**
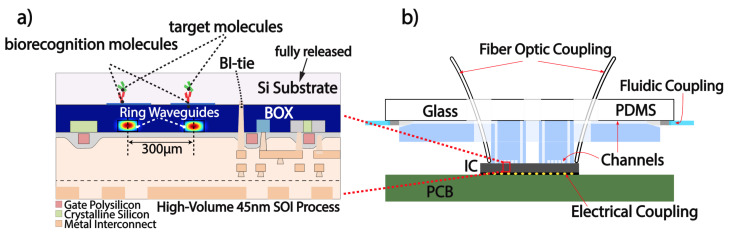
(**a**) CMOS 45RFSOI process cross-section. Backside Si substrate etch exposes the sensing photonics to the fluidic samples. (**b**) Cross section of microfluidic package aligned to chip. Photonic ports allow for fiber optic coupling, needles inserted at the edge allow for fluidic coupling, and flip chip bonding allows for electrical coupling. Layer 1 interfaces with the chip, layer 2 routes fluid to tubing, and layer 3 provides mechanical support.

Three-dimensional (3D) printed transfer molding enables rapid fabrication of multilayer microfluidics for co-packaging with highly miniaturized silicon photonic chips, as seen in Figure 1.

This versatile packaging technique can accommodate multi-fluidic and photonic coupling, independent of the electronic interface. As a first step, the advanced process node of the 45 nm Silicon on Insulator (SOI) fully integrated electronic-photonic platform requires removal of the Si substrate to prevent leakage of the optical mode in the substrate and expose the photonic sensors to the fluidic samples, as illustrated in Figure 1a. The Si substrate of the silicon-photonic chip is fully removed via a XeF${}_{2}$ etch. Because XeF${}_{2}$ is highly selective to silicon over silicon dioxide (SiO${}_{2}$), the buried oxide layer (BOX), which is SiO${}_{2}$-based, acts as a natural etch stop. The XeF${}_{2}$ etch is performed in excess until the silicon is visually observed to be removed. In the package that is shown in Figure 1b, the silicon chip is flip chip bonded on a printed circuit board (PCB) for electrical coupling and multilayer microfluidics are mounted on top of the chip. Our microfluidic design consists of two polydimethylsiloxane (PDMS) layers and a glass substrate for mechanical stability. The layers are enumerated by proximity to the chip, wherein the small area of the PDMS layer in contact with the chip is layer 1 (“primary layer”), the larger layer for fluid routing is layer 2 (“secondary layer”), and finally the glass substrate is layer 3.

### 2.1. 3D Printed Mold Fabrication

**Figure 2:**
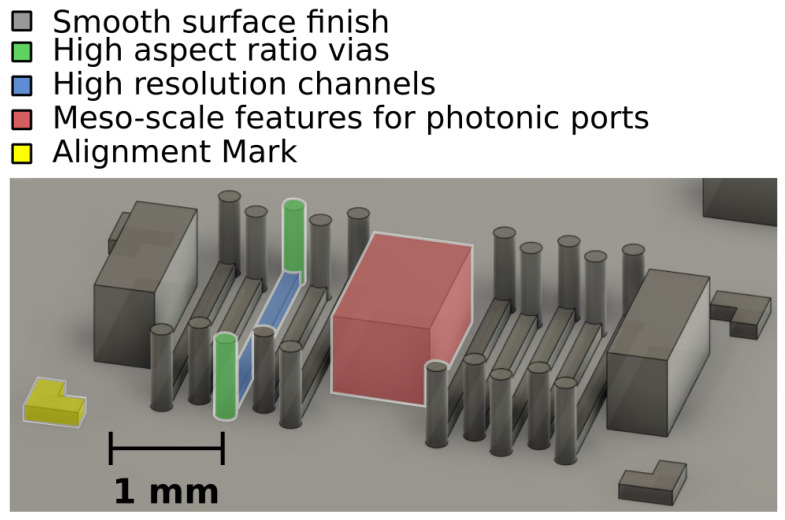
3D printed mold model for 10 channel device with 20 via-interconnections, 3 photonic ports, and alignment marks.

The mold for each PDMS layer is designed in CAD (Autodesk Fusion360) as an array of devices with various geometries of vertical interconnections (vias), channels, and access areas for optic fibers that act as photonic ports. We designed devices that range from four to 14 channels, eight to 28 vias, and three photonic ports on a 5.5 mm by 3 mm area to match the size of our sensing chip in order to test the limitations of the printed molds. The final 3D model forms interconnections with a height to width ratio between 5:1 to 10:1, high resolution channels, meso-scale photonic ports, and alignment marks as demonstrated in Figure 2. Alignment marks aid in precisely locating multiple layers while assembling the final device.

The model was submitted for fabrication by projection micro-stereolithography (Boston Micro Fabrication) with 2 μm resolution and using UV curable acrylate-based resins as the material. Bottom layer molds with varying channel widths were fabricated, achieving a smooth surface finish with interconnections of 5:1 to 10:1 height to width ratio, millimeter scale photonic ports, and an edge to edge channel spacing ranging from 150 μm to 200 μm, thus meeting the requirements for on-chip silicon photonic integration. The channel pitch was fixed at 300 μm set by the micro-ring resonator pitch, and the channel width was varied to assess risk of channel to channel leakage. Earlier attempts of printing similar molds while using high resolution fused deposition modeling and stereolithography printers failed to produce the necessary surface finish. Meanwhile, extremely high resolution 2 photon process printers could not produce larger meso-scale features.

### 2.2. Microfluidic Device Casting

**Figure 3:**
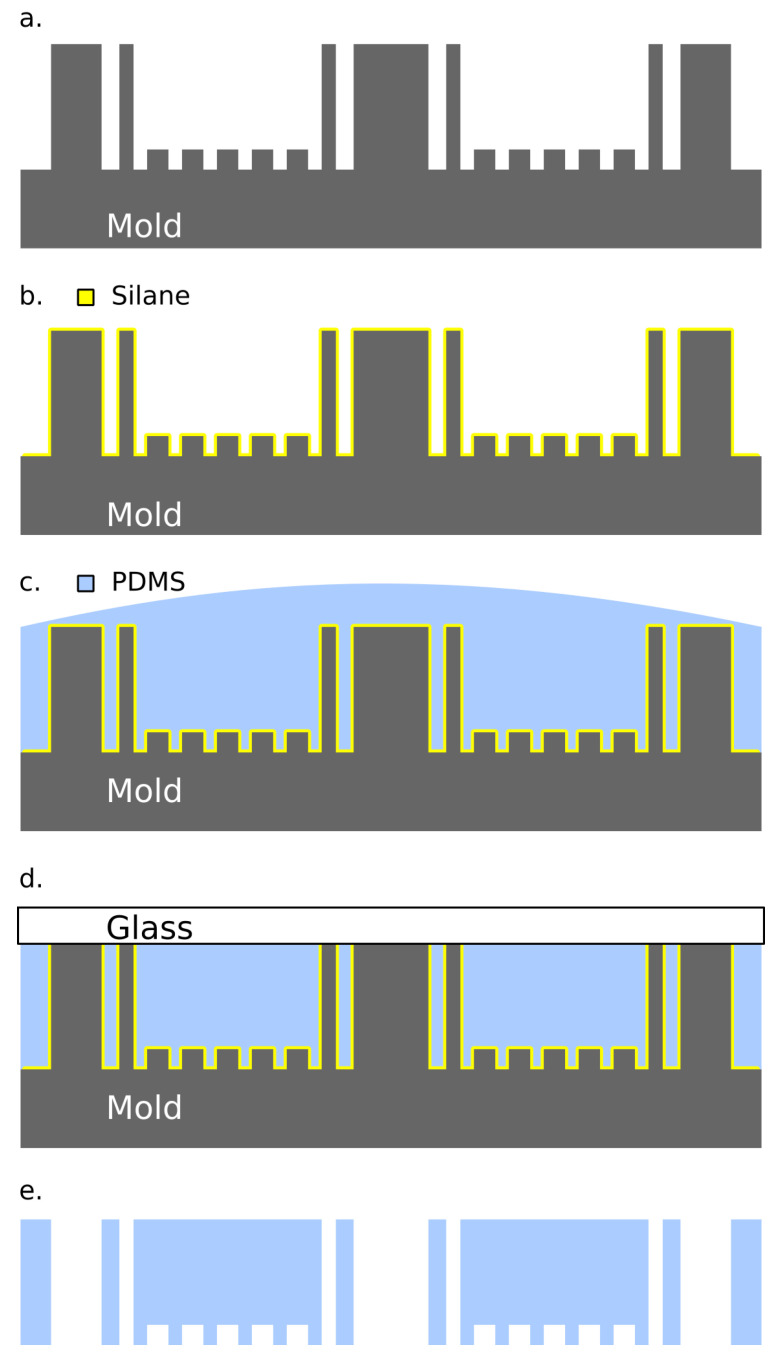
Workflow for casting polydimethylsiloxane (PDMS) on the three-dimensional (3D) printed mold. (**a**) The mold is (**b**) silanized in a vacuum chamber. (**c**) Mixed PDMS is poured onto the mold and (**d**) the top surface is made uniform with a glass cap. (**e**) The PDMS is then cured and removed from the mold.

The mold must be coated in a release agent prior to casting for a high-fidelity transfer of features without damage to delicate components of both the mold and the cast. To achieve this, the mold (Figure 3a) is placed in a vacuum chamber with 500 μL of Trichloro(1H, 1H, 2H, 2H-perfluorooctyl)silane (Sigma) under vacuum for 15 min. [19], as shown in Figure 3b. PDMS (Sylgard 184, Dow) is a composite of two materials: a pre-polymer, or material that can form into a polymer, and a crosslinking agent, which, when mixed with the pre-polymer, connects the molecules in a network that forms an elastic substrate. The pre-polymer and crosslinker are thoroughly mixed at the manufacturer’s recommended 10 parts pre-polymer to one part crosslinker by weight and degassed under vacuum (15 mbar) for 45 min. [20]. This mixture is poured onto the silanized mold (Figure 3c) and it cures into an elastic substrate that is patterned with the mold’s features. The mold is capped with a cleaned glass slide (Figure 3d) to ensure an even top surface with smooth finish required for multilayer bonding.

A 500-gram weight is then placed on top of the glass to remove residual PDMS between contact areas on the mold and glass in order assure thru-hole formation for vertical interconnections and photonic ports rather than thin membranes. The mold, PDMS, and glass sandwich are placed on a hot-plate at 95 degrees Celsius for 2 h to cure the elastomer. After the PDMS has cured, the cast device is removed from the device and then trimmed with a razor to its final size (Figure 3e). This process is repeated for each layer of the final multilayer device.

### 2.3. Alignment and Mechanical Sealing

**Figure 4:**
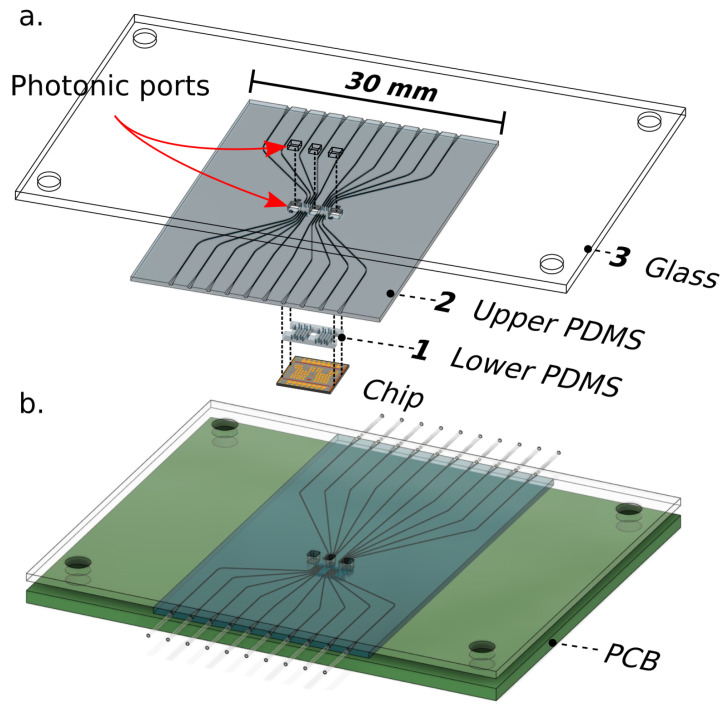
(**a**) Multilayer stack of glass substrate and cast PDMS pieces are aligned and then bonded using oxygen plasma. (**b**) The assembly is then coarsely aligned to the printed circuit board (PCB) before fine alignment of channels to sensing regions.

Once layers 1 and 2 have been cast, a glass substrate with milled photonic ports (layer 3) is prepared in order to provide mechanical support to the microfluidic channel networks. Layer 2, denoted as upper PDMS in Figure 4a, is first bonded to layer 3 with oxygen plasma by placing them in a reactive ion etch chamber with the mating surfaces exposed. Plasma introduces reactive hydroxyl groups on the surface that facilitate irreversible chemical bonding [21]. The mating surfaces are then aligned under magnification to maximize overlap between the photonic ports of the glass substrate and PDMS layer.

This assembly is placed on a hot plate at 65 degrees Celsius for 15 minutes to drive the reaction to completion. This process is repeated for bonding layer 1 to the layers 2,3 assembly. Coarse alignment is achieved by aligning corner holes in the glass substrate (Figure 4b) with holes on the PCB with screws. Fine alignment is further manually achieved by leveraging optical transparency through layers of the microfluidic stack for visual alignment of channels to sensing structures on-chip. Once aligned, the channels are sealed to the chip through mechanical pressure by tightening corner screws. Bonding the microfluidics to the chip exclusively through mechanical pressure allows for thorough decontamination of the chip surface between tests by entirely removing the package for reuse of the electronic-photonic chip. It also enables the rapid iteration of various channel geometries without requiring a unique chip to test each geometry.

## 3. Results and Discussions

### 3.1. Microfluidic Validation—Dye Test

**Figure 5:**
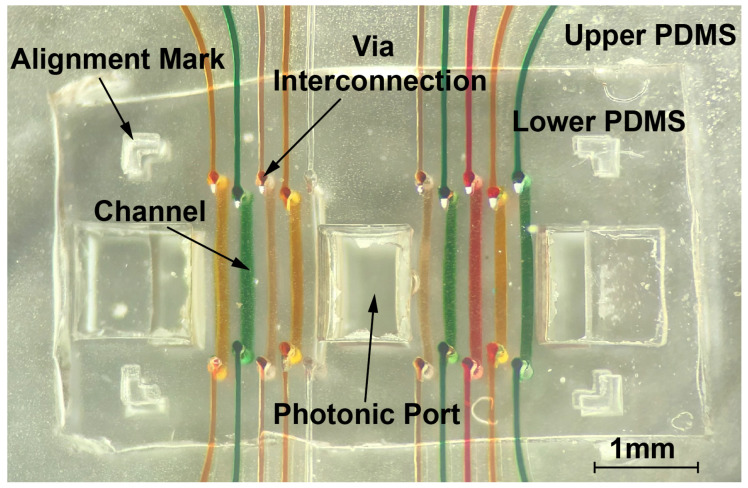
Colored dyes run through the multi-layer microfluidic assembly.

We demonstrate the functionality of the described microfluidic packaging by injecting colored dyes to detect leaks between channels. In Figure 5, colored dyes are injected into a 10-channel microfluidic device with 150 μm edge to edge channel spacing using edge coupling tubing with 28-gauge needles. One channel is kept empty as a reference. There was no cross contamination of dye in either the lower chip-sized PDMS channels or the larger upper PDMS channels. This test was done on glass for high contrast to best visualize any leaks. Once the proof of concept was verified while using dye on glass, a 10-channel microfluidic package with a 200 μm edge to edge channel spacing was aligned to 5 μm radius MRRs and optic fibers were aligned with grating couplers on-chip for photonic coupling.

### 3.2. Photonic Coupling and Bulk Sensitivity

**Figure 6:**
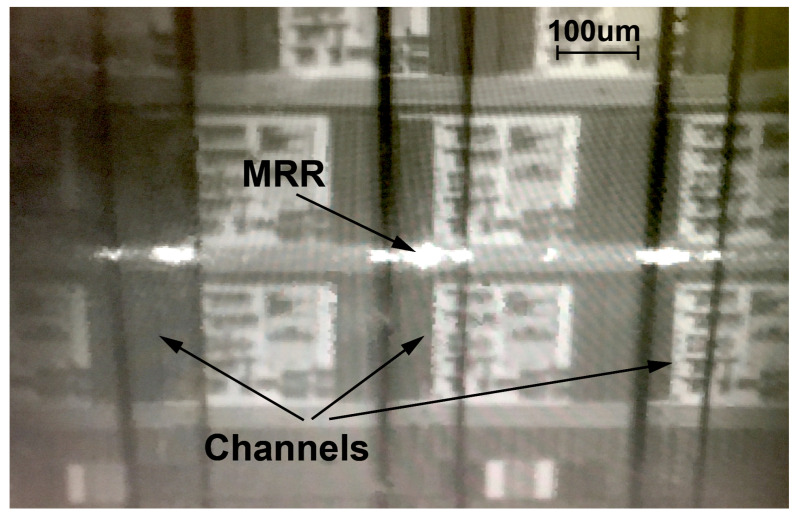
Power loss through micro-ring resonators while aligned to channels. This close up focuses on 3 channels from the 10-channel package of Figure 4.

Channels of the assembly were aligned to rings, as described above. Subsequently, 125 μm diameter lensed fibers are lowered through the photonic ports while using micromanipulators on an optical table. These fibers are aligned to on-chip grating couplers to couple laser light into a waveguide. When the wavelength of the input light is at the resonant wavelength of an MRR, power couples into, and circulates within, the micro-ring resulting in a power loss at the output coupler [22]. Figure 6 is an infrared image of channels that are aligned to the MRRs of a fully integrated electronic-photonic system on-chip. Photonic coupling is demonstrated by the circulating power emanating from the micro-rings.

**Figure 7:**
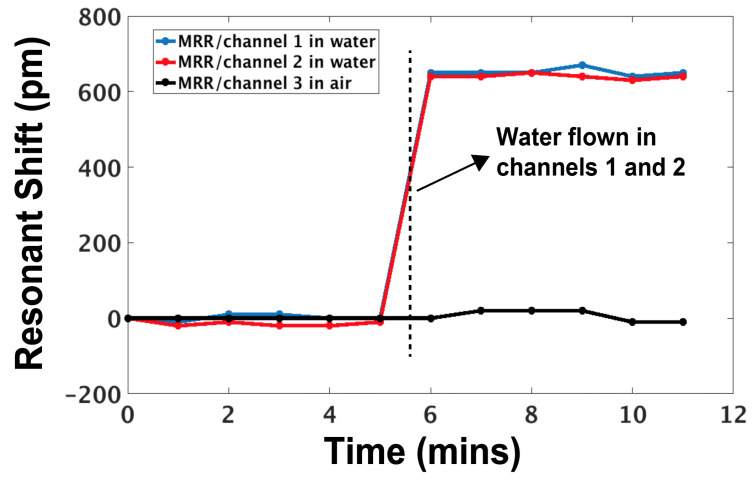
Resonant shift of micro-ring resonators (MRRs) exposed to water and air.

The bulk sensing capability of the platform was then evaluated using the proposed microfluidic packaging strategy. Light from a tunable laser (SANTEC TSL-510), which has the ability to change the wavelength of light output under fine control, is guided to a row of ring resonators. For these experiments, the light from the laser was swept from 1298 nm to 1308 nm. The laser performs multiple wavelength sweeps of the ring row’s output at a step of 10 picometers (pm). The output power is measured with a power meter (Agilent 8164B). For each sweep, the resonant wavelengths of the rings are captured and their difference from the nominal resonances of the initial sweep (resonant shift) is calculated. Figure 7 shows the resonant shift of three rings that were located in three different channels. Channels 1 and 2 are exposed to water, while channel 3 is empty. A resonant shift of 650pm was measured for the sensors under water. By characterizing the RI of water with an ATC refractometer (RI = 1.3334), a bulk sensitivity of 1.95 nm/Refractive Index Unit is calculated by dividing the resonant shift with the change of the RI from air to water (ΔRI=0.3334). This high channel density and miniaturized microfluidic device has the potential to integrate with a variety of silicon photonic devices.

## 4. Conclusions

Commercial projection micro stereolithography with sub 2 μm resolution paves the way for using three-dimensional (3D) printed transfer molding to create silicon photonic compatible microfluidics. The simultaneous success of fluidic, photonic, and electronic coupling using this rapid packaging strategy underlies its versatility. The facile fabrication of multilayer dense channel networks with smooth surface finish and photonic ports can solve key challenges for multi-fluid coupling, alignment of channels to micron scale sensing elements, and photonic coupling to the device. This unlocks the door towards compact and self-contained biophotonic sensors.
